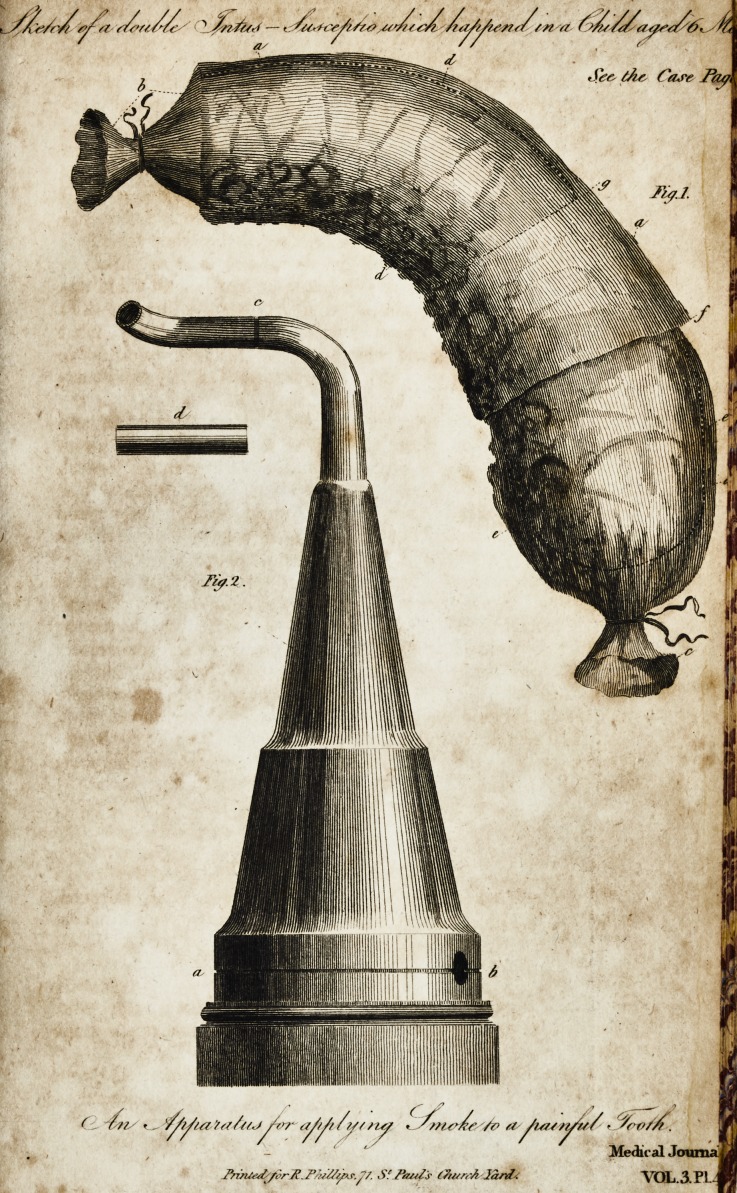# Mr. Spry, on Morbid Anatomy

**Published:** 1800-01

**Authors:** T. H. Spry

**Affiliations:** Surgeon. Aldersgate-street


### *To the Editors of the Medical and Phyfical Journal.*

GENTLEMEN,

WHEN I fent you the cafe of Hydrocephalus internus, I hinted in a curfory manner, the importance, and indeed neceffity, of anatomical inveftigation, in order to difcover the caufes of thofe difeafes of which we are ignorant; and, at the fame time, hinted the difficulties frequently attendant upon fuch examinations.

If it does not too much interfere with the plan of your Journal, I propofe to refume the fubject, and will endeavour to point out the importance of opening bodies after death; anatomy being the foundation upon which all true medical knowledge is built, and upon which, only, we can expect to raife a fabric which will ftand the teft of time.

Every practitioner in medicine will allow, that he has occafionally met with cafes which have completely baffled his moft ftrenuous endeavours to overcome the difeafe; and likewife his moft affiduous attempts to find out the caufe, which, if known on the firft attack of the diforder, might have faved him a great deal of unneceffary trouble and anxiety, and, in many inftances, the life of his patient. How mortifying muft it be to a practitioner, to find, upon examining the body of his patient after death, that he has been completely miftaken in his cafe; and that, probably, if he had known what his diforder really was in the firft inftance, a valuable life might have been faved!

We all know, that the fymptoms of many diforders are often equivocal, and that the confequence of this muft be, the medical attendant's occafionally miftaking one diforder for another; this is certainly a melancholy reflection, for, upon a proper, and indeed accurate diagnoftic, the patient's life frequently depends.

The moft fenfible and experienced are liable to thefe errors of judgment, as well as the illiterate and inexperienced; but, doubtlefs, they happen by far lefs often to the former than the latter; how careful then ought that man to be, who choofes the practice of phyfic or furgery for his employ, that he does not fport with the lives of his fellow-creatures.

The man who has the fcience of phyfic, rather than its forms and ceremonies, or craft, at heart, will be conftantly upon the watch to prevent accidents from happening in his practice, and will be daily improving himfelf, as well as rendering an effential fervice to others. Such a man as this may be juftly faid to be a valuable member of ſociety; while that man, whoſe only ſtudy it is to enrich himſelf at the expence of his fellow-creatures, by ſtudying the craft rather than the ſcience, and impoſing upon their credulity, may be conſidered as the peſt of ſociety.

By a more frequent examination of bodies after death, we may reaſonably hope that we ſhall in time acquire a fund of uſeful information, and be better able to avoid errors, as well as to adopt a more rational mode of practice in the treatment of thoſe diſeaſes, which are placed at preſent among the opprobia medicorum. When any thing extraordinary occurs, or any new diſcovery is made, ſuch occurrence or diſcovery ought moſt aſſuredly to be communicated to the public, with the particulars of the caſe (if poſſible); and, in ſome inſtances, a ſketch of the morbid part might be added, to illuſtrate the ſubject, and make it more comprehenſible, inſtead of hoarding up ſuch diſcoveries, which I am afraid is done too frequently, and only ſatisfies the ſelfiſh curioſity of the individual, without advancing our art in the ſmalleſt degree.

If ſuch a plan could be regularly practiſed, the phyſical and chirurgical arts would daily make more rapid ſtrides towards perfection than they do at preſent; although it muſt be conſeſſed, that very conſiderable improvements have lately been made in theſe ſciences, by a more general ſpirit of inveſtigation having taken place.

There is a number of diſorders to which the human body is liable, ſufficiently characterized by their peculiar ſymptoms; but the cauſes of many of them are veiled in obſcurity, having hitherto eluded the inquiries of medical men of the firſt abilities, nay, even of our beſt and moſt experienced anatomiſts. This, upon a ſuperficial view of the ſubject, is almoſt ſufficient to deter a perſon from proſecuting the matter further, and to make him reſt ſatisfied with what has been done before him, by practitioners whoſe abilities he conſiders in a far ſuperior degree to thoſe of his own, and to make him conclude, that if they have not been ſucceſsful, it is not at all probable that any inquiry he could make would be attended with better ſucceſs. Upon ſlightly conſidering the ſubject, ſome degree of plauſibility appears attached to it; yet, ſuch reaſoning as this is moſt aſſuredly very ſuperficial, and will, upon a more attentive examination, loſe its weight, and appear in the light it ought to do.

I would aſk, whether the immortal Hervey, when he began the ſtudy of anatomy, expected to diſcover the circulation? or Dr. Hunter the abſorbent ſyſtem? Moſt aſſuredly they did not; and yet we find that their anatomical ſtudies were crowned with a ſuca ſucceſs, far beyond their moſt ſanguine expectations, by the diſcoveries each of them made. A ſteady perſeverance, and a well-directed application to their purſuits, enabled them to do this: why may we not then expect, and with reaſonable hopes of our expectations ſucceeding, by purſuing a ſimilar plan, in a ſcience which preſents to our view ſuch an ample field for inveſtigation, that we may one day or other be equally ſucceſsful, and be able to point out the cauſes of ſome of thoſe diſeaſes at preſent unknown? How frequently do we ſee the difficulties which ariſe in the common occurrences of life, and which appear ſo formidable as to be thought almoſt, if not quite, inſurmountable, when preſented to our view, yield by courage and a ſteady conduct; and at laſt the difficulties, which had gradually diminiſhed, or loſt their terrors upon our nearer approach to them, diſappear altogether, fully recompenſing us for our exertions.

In this manner we ought to proceed in the ſciences of phyſic and ſurgery; difficulties will, no doubt, preſent themſelves daily, but, by their yielding to our well-applied exertions, we ſhall gain courage to perſevere, and, in time, what appears now ſo difficult to us, will no longer be ſeen in the ſame light.

We ſhould make it a rule attentively to examine the bodies of thoſe who die of diſorders unknown; or, ſuppoſing the diſeaſe to be known, yet the cauſe may be veiled in obſcurity; in either of theſe caſes there is ample ſcope for obſervation, and it would be hard indeed, if we did not ſometimes add a mite to our common ſtock of medical knowledge, by purſuing with vigour a plan of this kind.

By a ſteady perſeverance in the plan propoſed, whenever we have an opportunity, we ſhall gradually extend the boundaries of our art; if we ſhould be ſucceſsful in one caſe only, out of ten or more, yet it would amply repay us for all our trouble.

Men who wiſh well to the art will be daily taking pains to overcome the prejudices naturally inherent in the human mind againſt diſſecting bodies, and which exiſt in a much greater degree in thoſe people whoſe minds have not been cultivated by a liberal education, in order to obtain leave to examine the bodies of their patients after death.

The education of medical men being in general equal, and ſometimes ſuperior to that of others, they are often in conſequence much ſuperior in point of argument, and will therefore frequently be able to obtain their wiſhes, if they are ſtrenuous in their cauſe, by pointing out the abſurdity of the prejudices againſt opening bodies, and the great utility likely to be derived to poſterity, if ſuch examinations be permitted.

If conſent is obtained, no unneceſſary time ought to be loſt; but but it is advifable to do it almoft immediately, i. e. as foon as we can without giving offence to the friends or relations of the deceafed, by our betraying too great anxiety to begin our operation, as if we were void of feeling: a certain degree of folemnity is always prudent in thefe matters. It is well known to anatomifts, that the natural appearance of the different parts of the human body is foon changed after death; and that thofe parts which had been previoufly difeafed, fooner fuffer this change than thofe which had not, confequently they foon become very unfit for the inveftigation of the caufes of difeafes; for, after putrefaction has taken place in any confiderable degree, the appearance of the parts will be fo much altered, as to make it difficult for the moft experienced anatomift to fay, what parts had been difeafed and what had not: this is of confiderable importance, and ought conftantly to be attended to in our anatomical purfuits.

It was the opinion of a very juftly celebrated phyfician and anatomift, Dr. Wm. Hunter, that the moft probable means of improving the fcience of phyfic, would be "a more general, and a more accurate examination of difeafes after death." The opinion of fo great a man as Dr. Hunter, muft certainly add great weight to what I have faid; and if we are really defirous of promoting knowledge, it will have its proper effect.

Thus, Gentlemen, I have offered you my ideas on a fubject of no fmall importance; wifhing they may meet with your approbation and fupport. Having refolved, myfelf, to purfue the plan I have pointed out above, I fhall take every opportunity of communicating the refult of my inveftigations and inquiries to the public; thereby hoping to contribute my mite towards extending our art, as well as to improve myfelf.

I fhall fubjoin two curious cafes, which I had lately an opportunity of examining, together with a drawing to illuftrate one of them.

I remain, GENTLEMEN,

With refpect,

Your very humble Servant,

*Alderfgate-ftreet.* T. H. SPRY, Surgeon.

*Oct.* 1, 1799.

---

### CASE I.

The fubject of this cafe was a child, aged fix months, who, previoufly to the illnefs of which he died, was healthy and ftrong.

Upon infpecting the body, the attention was arrefted by the uncommon

Sketch of a double Intus – Susceptio which happend in a Child aged 6 M[illegible]

See the Case Pag[illegible]

An Apparatus for applying Smoke to a painful Tooth.

Printed for R. Phillips. 71. St. Paul's Church Yard.

uncommon diftention of the epigaftric and umbilical regions; the hypogaftric being nearly in its natural ftate.

Upon opening the abdomen, no omentum was to be feen; the tranfverfe arch of the colon was likewife miffing; the fmall inteftines were extremely diftended with air, and contained little fœculent matter; they did not appear to have been in the fmalleft degree inflamed.

On the left fide, near the loins, a very hard tumour, about four inches and a half in length, was foon difcovered.

Having made a couple of ligatures, one above and the other below the difeafed portion, I removed it, as reprefented in the plate: it appeared to be a double intus-fufceptio.

A portion of the inteftinum ilium, with the cæcum, its appendix, and the whole of the tranfverfe arch of the colon, had paffed downwards into the figmoid flexure; the cæcum, appendix cæci, with a fmall portion of the ilium, occupied the loweft part of the colon near its termination, and formed a firm mafs of a livid colour. A portion of the figmoid flexure was reflected over the tumour for the fpace of an inch, and had, by the ftricture it occcafioned, ftrangulated it: no adhefions had taken place. Between the fold of the lower intus-fufceptio, a quantity of a yellowifh coloured fluid was found.

The inteftine, when drawn out, meafured, from one ligature to the other, more than 22 inches, fo that about fixteen inches of inteftine had paffed from above downwards; and two inches from below upwards, at the lower intus-fufceptio. The information I received refpecting the fymptoms of this cafe was, that the child was firft feized with rigour, which was fucceeded by vomiting, and which continued to the laft. It had likewife one evacuation of pure blood, probably occafioned by the ftrangulation of the lower part of the tumor.

It is remarkable, that the child did not appear to fuffer the leaft pain. No medicine, as may be imagined, had the fmalleft effect.

The child was feized with rigour on Sunday the firft of September, and died on the Wednefday morning following;—and was opened the next morning.

From confidering the nature of this difeafe, and particularly from its fituation being near the rectum, which muft have been known, from the impoffibility of throwing more than a very fmall quantity into the rectum, which was actually the cafe, the clyfters being conftantly returned without paffing; does it not fuggeft the idea to us of making ufe of mechanical means, by the ufe of a large bougie introduced into the rectum? Would fuch practice be attended with any probability of fuccefs? or, would it be prudent to attempt it? Thefe are queftions which

I fhall

I ſhall leave to be decided by men of ſuperior abilities to my own; we are certain that death muſt be the conſequence, if this diſeaſe is left to itſelf, and that ſpeedily; are we not, on that account, authoriſed to try every means in our power, provided there is the ſmalleſt chance of ſuccefs?

### CASE II.

The ſubject of this caſe was a child aged twenty-two months. The whole body was emaciated to the greateſt degree poſſible, with anaſarcous extremities.

Upon opening the thorax, the lungs on the right ſide adhered to the pleura, lining the cavity of the thorax; but no mark of diſeaſe was perceived in the lungs themſelves. The pericardium was diſtended; and, upon opening it, a very large quantity of a reddiſh coloured fluid flowed out; the heart itſelf was ſound, but completely immerſed in fluid.

In the abdomen, the liver was larger than uſual, ſomewhat diſcoloured, and the left lobe was much indurated: nothing particular was perceived in the gall bladder and billiary ducts; the ſtomach was very large, and contained ſome indigeſtible matter; the inteſtines appeared in their natural ſtate: the meſentery ſhewed more evident marks of diſeaſe, being every where full of obſtructed glands, many large, being of the ſize of a walnut, and in ſome places they formed cluſters; the ſpleen, pancreas, and remainder of the abdominal viſcera were to appearance ſound.

This child, for ſix months after its birth, was to all appearance very healthy; about this time a purging came on, of a greeniſh, fœtid matter, which continued with greater or leſs violence till its death.

The child gradually waſted away, without any other ſymptom of diſeaſe beſides the diarrhœa; at firſt a vomiting attended the purging, but this, after a time, ceaſed.

She did not appear to ſuffer any particular pain, but was always languid; her appetite was frequently voracious.

She had ſometimes a cough, and, towards the laſt, her breathing was very quick; ſhe was reſtleſs, and would ſcream violently when ſhe awoke from her ſleep. Some little time before her death ſhe became quite anaſarcous; her extremities were diſtended to ſuch a degree as to threaten gangrene, the ſkin being tenſe, ſhining, and diſcoloured.

From the uniformity of the ſymptoms of this diſeaſe, for ſo great a length of time, no journal was kept of them, particularly as no medicines were exhibited, except at the beginning of the diſeaſe, when the purging came on.

### Explanation of Plate I. Vol. III. Fig. 1.

A. A. A. The colon at the figmoid flexure.
B. Inteftinum ilium paffing into the colon, and cut off above the ligature.
C. Colon cut off at its junction with the rectum.
D. D. Dotted lines, denoting the arch of the colon which had paffed into the figmoid flexure, altering the natural fhape of the latter.
E. E. Lines denoting a hard ftrangulated mafs in the loweft part of the figmoid flexure of the colon, formed by a portion of the ilium cæcum with its appendix, and part of the colon.
F. Intus-fufceptio formed by a portion of the figmoid flexure paffing upwards, and ftrangulating the tumour below it.
G. Extent of the lower intus-fufceptio.

## Figures and Tables

**Fig. 1. f1:**